# 2,8,15,18,21,24,31,37,44,47,50,53-Dodeca­oxahepta­cyclo­[52.4.0.0^4,35^.0^6,33^.0^9,14^.0^25,30^.0^38,43^]octa­penta­conta-1(54),4,6(33),9(14),10,12,25(30),26,28,34,38(43),39,41,55,57-penta­deca­ene di­chloro­methane disolvate

**DOI:** 10.1107/S1600536814007971

**Published:** 2014-04-16

**Authors:** Ji Ye Yun, Sung Wan Ahn, Dong Hwan Kim, Wonbo Sim, Jai Young Lee

**Affiliations:** a Department of Chemistry, Konyang University, Nonsan 320-711, Republic of Korea

## Abstract

In the title compound, C_46_H_50_O_12_·2CH_2_Cl_2_, each dual 20-crown-6 unit crystallizes with two di­chloro­methane solvent mol­ecules. The crown unit mol­ecule lies about an inversion centre located at the central benzene ring. The two crown ring groups adopt an *anti* conformation, stabilized by weak intra­molecular C—H⋯O inter­actions. In the crystal, the crown unit mol­ecules and the solvent mol­ecules are linked by C—H⋯O inter­actions into a three-dimensional network.

## Related literature   

For the preparation and crystal structures of related compounds, see: Lee *et al.* (2009 ▶); Beack *et al.* (2012 ▶). For background to dual crown ethers and their inclusion behavior, see: Lee *et al.* (1992 ▶, 1997 ▶).
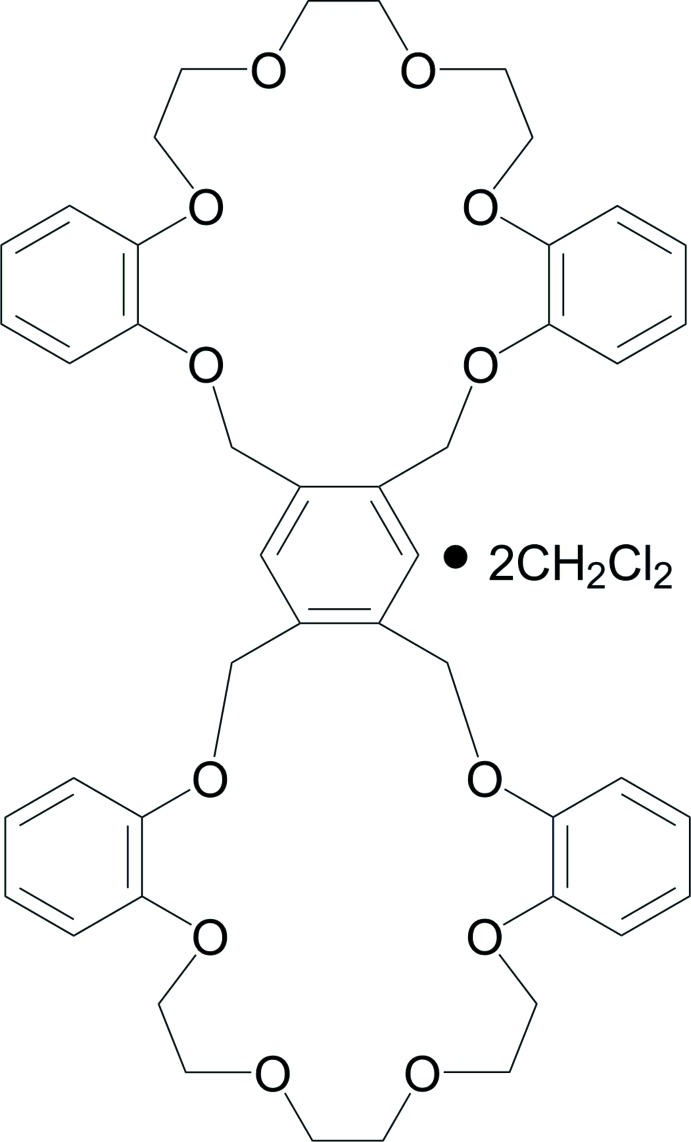


## Experimental   

### 

#### Crystal data  

C_46_H_50_O_12_·2CH_2_Cl_2_
*M* *_r_* = 964.71
Orthorhombic, 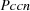

*a* = 23.0916 (11) Å
*b* = 23.9520 (11) Å
*c* = 8.6426 (4) Å
*V* = 4780.1 (4) Å^3^
*Z* = 4
Mo *K*α radiation
μ = 0.31 mm^−1^
*T* = 200 K
0.25 × 0.24 × 0.18 mm

#### Data collection  

Bruker APEXII CCD diffractometer
Absorption correction: multi-scan (*SADABS*; Bruker, 2008 ▶) *T*_min_ = 0.927, *T*_max_ = 0.947
26165 measured reflections
4211 independent reflections
2588 reflections with *I* > 2σ(*I*)
*R*_int_ = 0.077

#### Refinement  

*R*[*F* ^2^ > 2σ(*F* ^2^)] = 0.051
*wR*(*F* ^2^) = 0.136
*S* = 1.02
4211 reflections
289 parameters
H-atom parameters constrained
Δρ_max_ = 0.48 e Å^−3^
Δρ_min_ = −0.41 e Å^−3^

### 

Data collection: *APEX2* (Bruker, 2008 ▶); cell refinement: *SAINT-Plus* (Bruker, 2008 ▶); data reduction: *SAINT-Plus*; program(s) used to solve structure: *SHELXTL* (Sheldrick, 2008 ▶); program(s) used to refine structure: *SHELXTL*; molecular graphics: *SHELXTL*; software used to prepare material for publication: *SHELXTL*.

## Supplementary Material

Crystal structure: contains datablock(s) I, New_Global_Publ_Block. DOI: 10.1107/S1600536814007971/wm5018sup1.cif

Structure factors: contains datablock(s) I. DOI: 10.1107/S1600536814007971/wm5018Isup2.hkl

CCDC reference: 996417

Additional supporting information:  crystallographic information; 3D view; checkCIF report

## Figures and Tables

**Table 1:** Hydrogen-bond geometry (Å, °)

*D*—H⋯*A*	*D*—H	H⋯*A*	*D*⋯*A*	*D*—H⋯*A*
C1—H1*A*⋯O6^i^	0.95	2.47	2.828 (3)	103
C3—H3*B*⋯O4^ii^	0.99	2.59	3.457 (4)	146
C22—H22*A*⋯O1^ii^	0.99	2.32	3.252 (3)	156
C22—H22*B*⋯O1	0.99	2.53	2.909 (3)	103
C24—H24*A*⋯O6	0.99	2.51	3.455 (4)	160
C24—H24*B*⋯O3	0.99	2.36	3.331 (5)	168

Symmetry codes: (i) 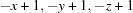; (ii) 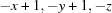.

**Fig. 1.**
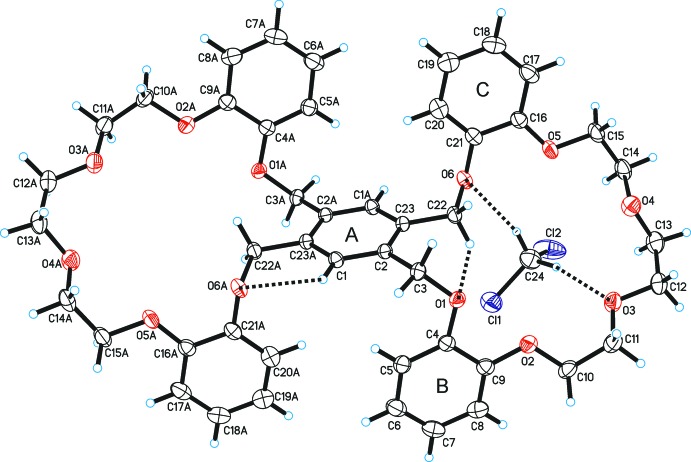
The molecular entities of the title compound with the atom numbering scheme and C—H···O interactions (dotted lines). Displacement ellipsoids are drawn at the 30% probability level. H atoms are presented as small spheres of arbitrary radius. [Symmetry code A): -x + 1, -y + 1, -z + 1].

**Fig. 2.**
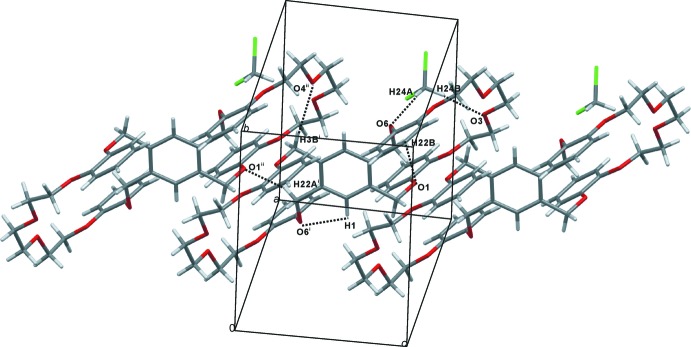
Crystal packing of the title compound with intra- and intermolecular C—H···O hydrogen bonds shown as dashed lines. [Symmetry codes: (i) -x + 1, -y + 1, -z + 1; (ii) -x + 1, -y + 1, -z.]

## supplementary crystallographic information

### 1. Chemical context

In a previous article we reported the synthesis and complexation behavior of common-nuclear bis-crown ethers (Lee *et al.*, 1992, 1997). Within this context we also reported the precursor of the common-nuclear bis­crown ether, bearing three aromatic subunits (Lee *et al.*, 2009; Beack *et al.*, 2012). The reaction of 1,2,4,5-tetra­kis(bromo­methyl)­benzene and bis­phenol in the presence of sodium hydride afforded the title compound, C_46_H_50_O_12_, that crystallizes with two molecules of di­chloro­methane.

### 2. Structural commentary

In the title molecule (Fig. 1), in the *A*-to-*B* ring and *A*-to-*C* ring connectivities, the torsion angles C2–C3–O1–C4 and C23–C22–O6–C21 are -72.7 (3) ° and 99.0 (3)°, respectively. This indicates that the *A* ring is situated *gauche* to the *B* ring, also situated *gauche* to the *C* ring, with dihedral angles of 81.64 (14)° between *A* and *B* and 81.16 (14)° between *A* and *C*. The dihedral angle between the *B* and *C* ring is 1.92 (15)°. All C–C–O–C torsion angles in the tri­ethyl­ene glycol group are related to *trans* conformations. Weak intra­molecular C—H···O hydrogen bonds (C22—H22B···O1; C1—H1A···O6) stabilize the conformation of the crown unit molecule (Fig. 1, Table 1).

### 3. Supra­molecular features

In the crystal, the crown unit molecules and the solvent molecules are linked by C—H···O inter­actions (Figs. 1, 2; Table 1). The C atom of the di­chloro­methane molecule (C24) is shifted by 2.383 Å outwards to the crown ring cavity (least squares plane defined by atoms O1–O6), and forms two C—H···O hydrogen bonds with two O atoms (O3 and O6) in the crown ring (Fig. 1, Table 1).

### 5. Synthesis and crystallization

To a refluxing suspension of sodium hydride (10.6 mmol) in THF under nitro­gen was added dropwise a solution of 1,2,4,5-tetra­kis(bromo­methyl)­benzene (2.20 mmol) and 1,8-bis­(2-hy­droxy­phen­oxy)-3,6-dioxao­ctane (4.40 mmol) in THF over a period of 3 h. The mixture was then refluxed for additional 3 days. After cooling to room temperature, 10%_wt_ aqueous hydro­chloric acid was added. The solvent was removed under reduced pressure and the residual mixture was extracted with di­chloro­methane. The organic layer was washed with water, dried over anhydrous magnesium sulfate, and evaporated *in vacuo*. The crude product was chromatographed on a silica-gel column using a mixed solvent of ethyl acetate and *n*-hexane (1:1) as eluent. Recrystallization from di­chloro­methane/*n*-hexane (1:20, v/v) resulted in 53% yield (m.p. 423 K). IR (KBr pellet): 2927, 1599, 1505, 1459, 1255, 1122, 1003, and 738 cm^-1^. ^1^H NMR (CDCl_3_): d 7.69 (s, 2 H, OCH_2_*Ar*), 6.92~6.80 (m, 16 H, O*Ar*O), 5.29 (s, 8 H, OC*H*_2_Ar), 4.12 (t, 8 H, ArOC*H*_2_CH_2_), 3.81 (t, 8 H, ArOCH_2_C*H*_2_) and 3.66 (t, 8 H, ArOCH_2_CH_2_OC*H*_2_).

### 6. Refinement details

All H-atoms were positioned geometrically and refined using a riding model with d(C—H)=0.95 Å, *U*_iso_=1.2*U*_eq_(C) for aromatic and 0.99 Å, *U*_iso_=1.2*U*_eq_(C) for CH_2_ atoms. Reflection (110) was affected by the beamstop and was omitted from the refinement.

### Crystal data

**Table:**

C_46_H_50_O_12_·2CH_2_Cl_2_	*F*(000) = 2024
*M**_r_* = 964.71	*D*_x_ = 1.340 Mg m^−^^3^
Orthorhombic, *P**c**c**n*	Mo *K*α radiation, λ = 0.71073 Å
Hall symbol: -P 2ab 2ac	Cell parameters from 4261 reflections
*a* = 23.0916 (11) Å	θ = 2.5–22.2°
*b* = 23.9520 (11) Å	µ = 0.31 mm^−^^1^
*c* = 8.6426 (4) Å	*T* = 200 K
*V* = 4780.1 (4) Å^3^	Block, colorless
*Z* = 4	0.25 × 0.24 × 0.18 mm

### Data collection

**Table:**

Bruker APEXII CCD diffractometer	4211 independent reflections
Radiation source: fine-focus sealed tube	2588 reflections with *I* > 2σ(*I*)
Graphite monochromator	*R*_int_ = 0.077
phi and ω scans	θ_max_ = 25.0°, θ_min_ = 2.5°
Absorption correction: multi-scan (*SADABS*; Bruker, 2008)	*h* = −27→22
*T*_min_ = 0.927, *T*_max_ = 0.947	*k* = −28→27
26165 measured reflections	*l* = −10→10

### Refinement

**Table:**

Refinement on *F*^2^	Primary atom site location: structure-invariant direct methods
Least-squares matrix: full	Secondary atom site location: difference Fourier map
*R*[*F*^2^ > 2σ(*F*^2^)] = 0.051	Hydrogen site location: inferred from neighbouring sites
*wR*(*F*^2^) = 0.136	H-atom parameters constrained
*S* = 1.02	*w* = 1/[σ^2^(*F*_o_^2^) + (0.0524*P*)^2^ + 2.3252*P*] where *P* = (*F*_o_^2^ + 2*F*_c_^2^)/3
4211 reflections	(Δ/σ)_max_ = 0.001
289 parameters	Δρ_max_ = 0.48 e Å^−^^3^
0 restraints	Δρ_min_ = −0.41 e Å^−^^3^

### Special details

**Table:**

Geometry. All e.s.d.'s (except the e.s.d. in the dihedral angle between two l.s. planes) are estimated using the full covariance matrix. The cell e.s.d.'s are taken into account individually in the estimation of e.s.d.'s in distances, angles and torsion angles; correlations between e.s.d.'s in cell parameters are only used when they are defined by crystal symmetry. An approximate (isotropic) treatment of cell e.s.d.'s is used for estimating e.s.d.'s involving l.s. planes.
Refinement. Refinement of *F*^2^ against ALL reflections. The weighted *R*-factor *wR* and goodness of fit *S* are based on *F*^2^, conventional *R*-factors *R* are based on *F*, with *F* set to zero for negative *F*^2^. The threshold expression of *F*^2^ > σ(*F*^2^) is used only for calculating *R*-factors(gt) *etc*. and is not relevant to the choice of reflections for refinement. *R*-factors based on *F*^2^ are statistically about twice as large as those based on *F*, and *R*- factors based on ALL data will be even larger.

### Fractional atomic coordinates and isotropic or equivalent isotropic displacement parameters (Å^2^)

**Table:**

	*x*	*y*	*z*	*U*_iso_*/*U*_eq_	
O1	0.54545 (8)	0.54550 (8)	0.1042 (2)	0.0372 (5)	
O2	0.63386 (9)	0.52751 (8)	−0.0718 (2)	0.0451 (5)	
O3	0.65434 (10)	0.42923 (8)	−0.2639 (2)	0.0527 (6)	
O4	0.58731 (10)	0.32856 (8)	−0.2708 (2)	0.0542 (6)	
O5	0.51826 (10)	0.31134 (8)	−0.0013 (2)	0.0492 (6)	
O6	0.52007 (9)	0.37487 (7)	0.2487 (2)	0.0405 (5)	
C1	0.48858 (12)	0.55573 (11)	0.4876 (3)	0.0315 (7)	
H1A	0.4806	0.5945	0.4790	0.038*	
C2	0.49824 (11)	0.52536 (11)	0.3538 (3)	0.0300 (6)	
C3	0.49439 (12)	0.55343 (12)	0.1976 (3)	0.0353 (7)	
H3A	0.4604	0.5385	0.1413	0.042*	
H3B	0.4881	0.5939	0.2131	0.042*	
C4	0.59462 (13)	0.57372 (11)	0.1441 (3)	0.0353 (7)	
C5	0.59937 (14)	0.61083 (12)	0.2657 (3)	0.0434 (8)	
H5A	0.5667	0.6184	0.3290	0.052*	
C6	0.65181 (16)	0.63729 (13)	0.2960 (4)	0.0532 (9)	
H6A	0.6548	0.6628	0.3799	0.064*	
C7	0.69873 (15)	0.62667 (13)	0.2057 (4)	0.0567 (9)	
H7A	0.7345	0.6446	0.2275	0.068*	
C8	0.69505 (14)	0.58999 (13)	0.0822 (4)	0.0515 (9)	
H8A	0.7281	0.5830	0.0198	0.062*	
C9	0.64311 (13)	0.56349 (11)	0.0498 (3)	0.0398 (7)	
C10	0.68141 (14)	0.52010 (13)	−0.1761 (4)	0.0494 (8)	
H10A	0.7139	0.5015	−0.1219	0.059*	
H10B	0.6951	0.5569	−0.2133	0.059*	
C11	0.66245 (15)	0.48529 (13)	−0.3103 (4)	0.0530 (9)	
H11A	0.6257	0.5003	−0.3525	0.064*	
H11B	0.6921	0.4870	−0.3929	0.064*	
C12	0.65282 (15)	0.39212 (13)	−0.3923 (4)	0.0527 (9)	
H12A	0.6898	0.3944	−0.4502	0.063*	
H12B	0.6210	0.4028	−0.4631	0.063*	
C13	0.64360 (15)	0.33379 (14)	−0.3356 (4)	0.0572 (9)	
H13A	0.6480	0.3072	−0.4226	0.069*	
H13B	0.6731	0.3246	−0.2563	0.069*	
C14	0.57832 (16)	0.27550 (12)	−0.2012 (4)	0.0555 (9)	
H14A	0.6080	0.2690	−0.1205	0.067*	
H14B	0.5820	0.2458	−0.2802	0.067*	
C15	0.51951 (16)	0.27369 (13)	−0.1309 (4)	0.0539 (9)	
H15A	0.4901	0.2849	−0.2083	0.065*	
H15B	0.5106	0.2353	−0.0961	0.065*	
C16	0.46768 (14)	0.31477 (12)	0.0792 (3)	0.0408 (7)	
C17	0.41664 (16)	0.28668 (12)	0.0421 (4)	0.0525 (9)	
H17A	0.4157	0.2623	−0.0446	0.063*	
C18	0.36749 (16)	0.29396 (13)	0.1303 (4)	0.0557 (9)	
H18A	0.3328	0.2751	0.1032	0.067*	
C19	0.36849 (15)	0.32842 (14)	0.2573 (4)	0.0554 (9)	
H19A	0.3347	0.3331	0.3185	0.066*	
C20	0.41895 (14)	0.35637 (13)	0.2958 (4)	0.0494 (8)	
H20A	0.4195	0.3805	0.3831	0.059*	
C21	0.46838 (13)	0.34958 (11)	0.2089 (3)	0.0381 (7)	
C22	0.52138 (13)	0.43413 (11)	0.2221 (3)	0.0384 (7)	
H22A	0.4919	0.4437	0.1431	0.046*	
H22B	0.5598	0.4444	0.1801	0.046*	
C23	0.50998 (12)	0.46813 (11)	0.3662 (3)	0.0309 (7)	
C24	0.65709 (16)	0.37292 (13)	0.0881 (4)	0.0657 (10)	
H24A	0.6180	0.3635	0.1271	0.079*	
H24B	0.6532	0.3846	−0.0213	0.079*	
Cl1	0.68538 (5)	0.42883 (4)	0.19717 (12)	0.0760 (3)	
Cl2	0.70129 (5)	0.31375 (4)	0.09828 (17)	0.1023 (5)	

### Atomic displacement parameters (Å^2^)

**Table:**

	*U*^11^	*U*^22^	*U*^33^	*U*^12^	*U*^13^	*U*^23^
O1	0.0369 (12)	0.0466 (12)	0.0280 (10)	−0.0047 (9)	0.0004 (9)	0.0016 (9)
O2	0.0423 (13)	0.0467 (12)	0.0462 (12)	−0.0017 (10)	0.0089 (10)	−0.0019 (10)
O3	0.0675 (16)	0.0459 (13)	0.0448 (13)	0.0044 (11)	0.0116 (11)	−0.0022 (10)
O4	0.0609 (16)	0.0430 (13)	0.0586 (14)	0.0097 (11)	0.0076 (12)	−0.0021 (11)
O5	0.0646 (16)	0.0397 (12)	0.0433 (13)	−0.0064 (11)	0.0002 (11)	−0.0150 (10)
O6	0.0478 (13)	0.0330 (11)	0.0407 (12)	0.0052 (9)	−0.0076 (10)	−0.0096 (9)
C1	0.0347 (17)	0.0285 (14)	0.0314 (16)	0.0019 (12)	−0.0023 (13)	−0.0001 (12)
C2	0.0309 (16)	0.0350 (15)	0.0242 (15)	−0.0015 (12)	−0.0018 (12)	0.0008 (12)
C3	0.0363 (18)	0.0404 (16)	0.0293 (15)	0.0018 (13)	0.0005 (13)	0.0006 (13)
C4	0.0412 (18)	0.0330 (15)	0.0318 (16)	−0.0015 (13)	−0.0045 (14)	0.0070 (13)
C5	0.049 (2)	0.0461 (18)	0.0356 (17)	−0.0046 (15)	−0.0006 (15)	0.0023 (14)
C6	0.062 (2)	0.0460 (19)	0.052 (2)	−0.0118 (17)	−0.0069 (18)	−0.0019 (16)
C7	0.046 (2)	0.053 (2)	0.070 (2)	−0.0133 (17)	−0.0107 (19)	−0.0001 (19)
C8	0.041 (2)	0.0488 (19)	0.064 (2)	−0.0030 (16)	0.0037 (17)	0.0056 (17)
C9	0.046 (2)	0.0339 (16)	0.0397 (17)	0.0005 (14)	−0.0002 (15)	0.0047 (14)
C10	0.046 (2)	0.0490 (19)	0.054 (2)	0.0010 (16)	0.0166 (16)	0.0026 (17)
C11	0.056 (2)	0.049 (2)	0.053 (2)	0.0049 (17)	0.0181 (17)	0.0034 (17)
C12	0.054 (2)	0.060 (2)	0.0443 (19)	0.0097 (17)	0.0051 (16)	−0.0080 (17)
C13	0.058 (2)	0.054 (2)	0.060 (2)	0.0160 (17)	0.0065 (19)	−0.0147 (18)
C14	0.077 (3)	0.0380 (18)	0.051 (2)	0.0061 (17)	−0.0004 (19)	−0.0140 (16)
C15	0.077 (3)	0.0376 (17)	0.0471 (19)	−0.0057 (17)	−0.0002 (19)	−0.0130 (15)
C16	0.051 (2)	0.0325 (16)	0.0385 (17)	−0.0021 (15)	−0.0043 (16)	0.0021 (14)
C17	0.071 (3)	0.0365 (18)	0.050 (2)	−0.0106 (17)	−0.0112 (19)	−0.0001 (15)
C18	0.052 (2)	0.047 (2)	0.068 (2)	−0.0098 (17)	−0.014 (2)	0.0106 (19)
C19	0.049 (2)	0.058 (2)	0.059 (2)	0.0033 (17)	−0.0062 (18)	0.0075 (19)
C20	0.052 (2)	0.0482 (19)	0.047 (2)	0.0087 (17)	−0.0097 (17)	−0.0010 (16)
C21	0.046 (2)	0.0294 (15)	0.0389 (17)	0.0039 (14)	−0.0078 (15)	0.0012 (14)
C22	0.0486 (19)	0.0345 (16)	0.0322 (16)	−0.0012 (14)	0.0008 (14)	−0.0061 (13)
C23	0.0330 (17)	0.0345 (15)	0.0253 (15)	−0.0006 (12)	−0.0013 (12)	−0.0034 (12)
C24	0.065 (3)	0.061 (2)	0.071 (2)	−0.0013 (19)	−0.010 (2)	0.0017 (19)
Cl1	0.0777 (7)	0.0795 (7)	0.0707 (7)	−0.0151 (5)	−0.0071 (5)	−0.0053 (5)
Cl2	0.0663 (7)	0.0586 (6)	0.1820 (13)	−0.0014 (5)	−0.0233 (8)	0.0133 (7)

### Geometric parameters (Å, º)

**Table:**

O1—C4	1.366 (3)	C10—H10B	0.9900
O1—C3	1.441 (3)	C11—H11A	0.9900
O2—C9	1.376 (3)	C11—H11B	0.9900
O2—C10	1.432 (3)	C12—C13	1.496 (4)
O3—C11	1.414 (3)	C12—H12A	0.9900
O3—C12	1.423 (3)	C12—H12B	0.9900
O4—C13	1.421 (4)	C13—H13A	0.9900
O4—C14	1.421 (4)	C13—H13B	0.9900
O5—C16	1.362 (4)	C14—C15	1.488 (5)
O5—C15	1.438 (3)	C14—H14A	0.9900
O6—C21	1.382 (3)	C14—H14B	0.9900
O6—C22	1.438 (3)	C15—H15A	0.9900
C1—C2	1.384 (4)	C15—H15B	0.9900
C1—C23^i^	1.387 (4)	C16—C17	1.394 (4)
C1—H1A	0.9500	C16—C21	1.397 (4)
C2—C23	1.402 (3)	C17—C18	1.378 (5)
C2—C3	1.510 (4)	C17—H17A	0.9500
C3—H3A	0.9900	C18—C19	1.373 (5)
C3—H3B	0.9900	C18—H18A	0.9500
C4—C5	1.381 (4)	C19—C20	1.384 (4)
C4—C9	1.407 (4)	C19—H19A	0.9500
C5—C6	1.392 (4)	C20—C21	1.376 (4)
C5—H5A	0.9500	C20—H20A	0.9500
C6—C7	1.360 (5)	C22—C23	1.511 (4)
C6—H6A	0.9500	C22—H22A	0.9900
C7—C8	1.385 (4)	C22—H22B	0.9900
C7—H7A	0.9500	C23—C1^i^	1.387 (4)
C8—C9	1.386 (4)	C24—Cl2	1.749 (4)
C8—H8A	0.9500	C24—Cl1	1.763 (3)
C10—C11	1.494 (4)	C24—H24A	0.9900
C10—H10A	0.9900	C24—H24B	0.9900
			
C4—O1—C3	118.3 (2)	H12A—C12—H12B	108.3
C9—O2—C10	116.1 (2)	O4—C13—C12	110.0 (3)
C11—O3—C12	112.0 (2)	O4—C13—H13A	109.7
C13—O4—C14	112.3 (2)	C12—C13—H13A	109.7
C16—O5—C15	116.9 (2)	O4—C13—H13B	109.7
C21—O6—C22	114.3 (2)	C12—C13—H13B	109.7
C2—C1—C23^i^	122.7 (2)	H13A—C13—H13B	108.2
C2—C1—H1A	118.6	O4—C14—C15	109.4 (3)
C23^i^—C1—H1A	118.6	O4—C14—H14A	109.8
C1—C2—C23	118.7 (2)	C15—C14—H14A	109.8
C1—C2—C3	120.2 (2)	O4—C14—H14B	109.8
C23—C2—C3	121.0 (2)	C15—C14—H14B	109.8
O1—C3—C2	113.2 (2)	H14A—C14—H14B	108.2
O1—C3—H3A	108.9	O5—C15—C14	108.5 (3)
C2—C3—H3A	108.9	O5—C15—H15A	110.0
O1—C3—H3B	108.9	C14—C15—H15A	110.0
C2—C3—H3B	108.9	O5—C15—H15B	110.0
H3A—C3—H3B	107.8	C14—C15—H15B	110.0
O1—C4—C5	125.2 (3)	H15A—C15—H15B	108.4
O1—C4—C9	115.4 (2)	O5—C16—C17	125.4 (3)
C5—C4—C9	119.3 (3)	O5—C16—C21	115.8 (3)
C4—C5—C6	120.4 (3)	C17—C16—C21	118.8 (3)
C4—C5—H5A	119.8	C18—C17—C16	120.5 (3)
C6—C5—H5A	119.8	C18—C17—H17A	119.7
C7—C6—C5	120.0 (3)	C16—C17—H17A	119.7
C7—C6—H6A	120.0	C19—C18—C17	120.3 (3)
C5—C6—H6A	120.0	C19—C18—H18A	119.9
C6—C7—C8	120.8 (3)	C17—C18—H18A	119.9
C6—C7—H7A	119.6	C18—C19—C20	119.8 (3)
C8—C7—H7A	119.6	C18—C19—H19A	120.1
C7—C8—C9	120.0 (3)	C20—C19—H19A	120.1
C7—C8—H8A	120.0	C21—C20—C19	120.6 (3)
C9—C8—H8A	120.0	C21—C20—H20A	119.7
O2—C9—C8	125.2 (3)	C19—C20—H20A	119.7
O2—C9—C4	115.4 (3)	C20—C21—O6	121.9 (3)
C8—C9—C4	119.5 (3)	C20—C21—C16	119.9 (3)
O2—C10—C11	109.5 (3)	O6—C21—C16	118.1 (3)
O2—C10—H10A	109.8	O6—C22—C23	113.4 (2)
C11—C10—H10A	109.8	O6—C22—H22A	108.9
O2—C10—H10B	109.8	C23—C22—H22A	108.9
C11—C10—H10B	109.8	O6—C22—H22B	108.9
H10A—C10—H10B	108.2	C23—C22—H22B	108.9
O3—C11—C10	110.4 (3)	H22A—C22—H22B	107.7
O3—C11—H11A	109.6	C1^i^—C23—C2	118.5 (2)
C10—C11—H11A	109.6	C1^i^—C23—C22	121.6 (2)
O3—C11—H11B	109.6	C2—C23—C22	119.8 (2)
C10—C11—H11B	109.6	Cl2—C24—Cl1	111.9 (2)
H11A—C11—H11B	108.1	Cl2—C24—H24A	109.2
O3—C12—C13	109.3 (3)	Cl1—C24—H24A	109.2
O3—C12—H12A	109.8	Cl2—C24—H24B	109.2
C13—C12—H12A	109.8	Cl1—C24—H24B	109.2
O3—C12—H12B	109.8	H24A—C24—H24B	107.9
C13—C12—H12B	109.8		
			
C23^i^—C1—C2—C23	0.0 (4)	C16—O5—C15—C14	−179.3 (3)
C23^i^—C1—C2—C3	178.2 (3)	O4—C14—C15—O5	−68.1 (3)
C4—O1—C3—C2	−72.7 (3)	C15—O5—C16—C17	−2.9 (4)
C1—C2—C3—O1	126.4 (3)	C15—O5—C16—C21	177.1 (2)
C23—C2—C3—O1	−55.4 (3)	O5—C16—C17—C18	−178.9 (3)
C3—O1—C4—C5	−1.8 (4)	C21—C16—C17—C18	1.2 (4)
C3—O1—C4—C9	179.1 (2)	C16—C17—C18—C19	−1.0 (5)
O1—C4—C5—C6	179.9 (3)	C17—C18—C19—C20	0.6 (5)
C9—C4—C5—C6	−0.9 (4)	C18—C19—C20—C21	−0.6 (5)
C4—C5—C6—C7	0.0 (5)	C19—C20—C21—O6	−176.9 (3)
C5—C6—C7—C8	0.5 (5)	C19—C20—C21—C16	0.9 (4)
C6—C7—C8—C9	−0.2 (5)	C22—O6—C21—C20	−72.7 (3)
C10—O2—C9—C8	−3.7 (4)	C22—O6—C21—C16	109.4 (3)
C10—O2—C9—C4	175.2 (2)	O5—C16—C21—C20	178.9 (3)
C7—C8—C9—O2	178.1 (3)	C17—C16—C21—C20	−1.1 (4)
C7—C8—C9—C4	−0.7 (4)	O5—C16—C21—O6	−3.2 (4)
O1—C4—C9—O2	1.5 (3)	C17—C16—C21—O6	176.7 (2)
C5—C4—C9—O2	−177.7 (2)	C21—O6—C22—C23	98.8 (3)
O1—C4—C9—C8	−179.5 (2)	C1—C2—C23—C1^i^	0.0 (4)
C5—C4—C9—C8	1.2 (4)	C3—C2—C23—C1^i^	−178.2 (3)
C9—O2—C10—C11	−173.6 (2)	C1—C2—C23—C22	−179.3 (3)
C12—O3—C11—C10	−163.0 (3)	C3—C2—C23—C22	2.5 (4)
O2—C10—C11—O3	−71.6 (3)	O6—C22—C23—C1^i^	13.6 (4)
C11—O3—C12—C13	−179.5 (3)	O6—C22—C23—C2	−167.1 (2)
C14—O4—C13—C12	−174.2 (3)	C2—C3—O1—C4	−72.7 (3)
O3—C12—C13—O4	67.2 (3)	C23—C22—O6—C21	98.8 (3)
C13—O4—C14—C15	177.9 (3)		

Symmetry code: (i) −*x*+1, −*y*+1, −*z*+1.

### Hydrogen-bond geometry (Å, º)

**Table:**

*D*—H···*A*	*D*—H	H···*A*	*D*···*A*	*D*—H···*A*
C1—H1*A*···O6^i^	0.95	2.47	2.828 (3)	103
C3—H3*B*···O4^ii^	0.99	2.59	3.457 (4)	146
C22—H22*A*···O1^ii^	0.99	2.32	3.252 (3)	156
C22—H22*B*···O1	0.99	2.53	2.909 (3)	103
C24—H24*A*···O6	0.99	2.51	3.455 (4)	160
C24—H24*B*···O3	0.99	2.36	3.331 (5)	168

Symmetry codes: (i) −*x*+1, −*y*+1, −*z*+1; (ii) −*x*+1, −*y*+1, −*z*.
